# Fate of adipocyte progenitors during adipogenesis in mice fed a high-fat diet

**DOI:** 10.1016/j.molmet.2021.101328

**Published:** 2021-09-23

**Authors:** Muhammad Bilal, Allah Nawaz, Tomonobu Kado, Muhammad Rahil Aslam, Yoshiko Igarashi, Ayumi Nishimura, Yoshiyuki Watanabe, Takahide Kuwano, Jianhui Liu, Hiroyuki Miwa, Takumi Era, Koichi Ikuta, Johji Imura, Kunimasa Yagi, Takashi Nakagawa, Shiho Fujisaka, Kazuyuki Tobe

**Affiliations:** 1 First Department of Internal Medicine, Faculty of Medicine, University of Toyama, 2630 Sugitani, Toyama, 930-0194, Japan; 2 Department of Molecular and Medical Pharmacology, Faculty of Medicine, University of Toyama, 2630 Sugitani, Toyama, 930-0194, Japan; 3 Department of Cell Modulation, Institute of Molecular Embryology and Genetics, Kumamoto University, 2-2-1 Honjo, Chuo-ku, Kumamoto, 860-0811, Japan; 4 Department of Virus Research, Laboratory of Immune Regulation, Institute of Frontier Life and Medical Sciences, Kyoto University, Sakyo-ku, Kyoto, 606-8507, Japan; 5 Department of Diagnostic Pathology, Faculty of Medicine, University of Toyama, 2630 Sugitani, Toyama, 930-0194, Japan

**Keywords:** Adipocyte hyperplasia, Obesity, Adipogenesis, Adipocyte progenitors

## Abstract

**Objective:**

Expansion of adipose tissue during obesity through the recruitment of newly generated adipocytes (hyperplasia) is metabolically healthy, whereas that through the enlargement of pre-existing adipocytes (hypertrophy) leads to metabolic complications. Accumulating evidence from genetic fate mapping studies suggests that in animal models receiving a high-fat diet (HFD), only adipocyte progenitors (APs) in gonadal white adipose tissue (gWAT) have proliferative potential. However, the proliferative potential and differentiating capacity of APs in the inguinal WAT (iWAT) of male mice remains controversial. The objective of this study was to investigate the proliferative and adipogenic potential of APs in the iWAT of HFD-fed male mice.

**Methods:**

We generated PDGFRα-GFP-Cre-ER^T2^/tdTomato (KI/td) mice and traced PDGFRα-positive APs in male mice fed HFD for 8 weeks. We performed a comprehensive phenotypic analysis, including the histology, immunohistochemistry, flow cytometry, and gene expression analysis, of KI/td mice fed HFD.

**Results:**

Contrary to the findings of others, we found an increased number of newly generated tdTomato^+^ adipocytes in the iWAT of male mice, which was smaller than that observed in the gWAT. We found that in male mice, the iWAT has more proliferating tdTomato^+^ APs than the gWAT. We also found that tdTomato^+^ APs showed a higher expression of *Dpp4* and *Pi16* than tdTomato^−^ APs, and the expression of these genes was significantly higher in the iWAT than in the gWAT of mice fed HFD for 8 weeks. Collectively, our results reveal that HFD feeding induces the proliferation of tdTomato^+^ APs in the iWAT of male mice.

**Conclusion:**

In male mice, compared with gWAT, iWAT undergoes hyperplasia in response to 8 weeks of HFD feeding through the recruitment of newly generated adipocytes due to an abundance of APs with a high potential for proliferation and differentiation.

## Highlights

• High-fat diet (HFD) increases proliferative APs in iWAT of male mice.
• HFD promotes the recruitment of smaller adipocytes in the iWAT of male mice.
• iWAT expands by recruiting newly-generated adipocytes (hyperplasia) in male mice.
• gWAT expands by enlargement of pre-existing adipocytes (hypertrophy) in male mice.

## Graphical abstract

**Figure:**
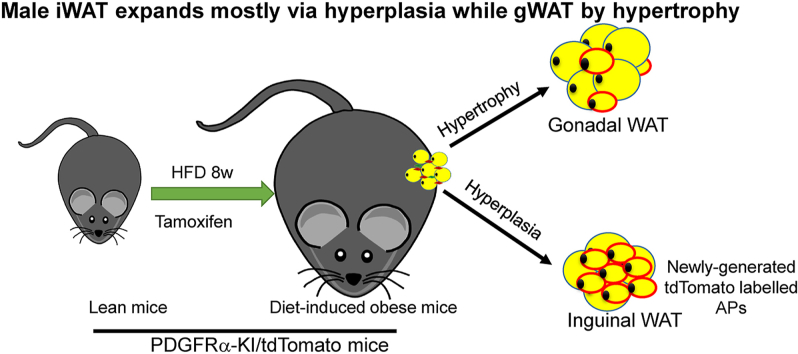

## Abbreviations

WAT: White Adipose Tissue
gWAT: Visceral or Gonadal WAT
eWAT: Epididymal WAT
iWAT: Subcutaneous or Inguinal WAT (iWAT)
APs: Adipocyte Progenitors
HFD: High Fat Diet
NC: Normal Chow
BW: Body Weight
PDGFRα: Platelet-derived Growth Factor Receptor-alpha
KI/+: PDGFRα-GFP-Cre-ER^T2^ knock-in
KI/td: PDGFRα-GFP-Cre-ER^T2^/tdTomato mice
TAM: Tamoxifen
FACS: Fluorescence-activated Cell Sorting
WT: Wild-type

## 1 Introduction

Obesity is characterized by the expansion of white adipose tissue (WAT) in response to HFD feeding, either by the enlargement of the existing adipocytes (hypertrophy) or through the recruitment of new adipocytes (hyperplasia) [[1], [2], [3], [4], [5]]. Several studies have shown that the imbalance between adipocyte hypertrophy and hyperplasia in WAT depots is associated with metabolic disturbances, including insulin resistance, cardiometabolic risk, dyslipidemia, and atherosclerosis [[4], [5], [6], [7], [8], [9], [10], [11], [12]].

WAT is mainly distributed in two major fat depots, namely visceral or gonadal WAT (gWAT) and subcutaneous or inguinal WAT (iWAT). To meet the energy balance during HFD intake, these depots can expand by up to 70% [[13], [14], [15]]. The expansion of iWAT is related to preserving insulin sensitivity, less inflammation and improving the metabolic profile, whereas gWAT expansion is associated with larger adipocytes and proinflammatory macrophages, leading to insulin resistance [[16], [17], [18], [19], [20], [21], [22]]. The expansion of WAT depots was found to occur in different patterns in males and females [6,23,24]. Joe et al. showed that only adipocyte progenitors (APs) in iWAT, and not in gWAT, exhibit proliferative potential in HFD-fed male mice [25]. In contrast, studies on lineage tracing using genetic mice models revealed that gWAT in adult male mice expands through adipocyte hyperplasia and hypertrophy, whereas iWAT expands exclusively through cellular hypertrophy under HFD-fed conditions [5,6,[26], [27], [28]]. How individual adipose depots expand by hypertrophy or hyperplasia, especially in HFD-fed male mice, is unknown.

Platelet-derived growth factor receptor-alpha (PDGFRα), a membrane-bound tyrosine kinase receptor that is expressed in different tissues during embryogenesis, is an ideal marker of APs [29]. Recent reports on single-cell RNA-sequencing analysis have revealed that WAT contains at least two major subtypes of APs, namely DPP4^+^ APs and ICAM-1^+^ APs. DPP4^+^ APs are highly proliferative, whereas ICAM-1^+^ APs are committed to an adipogenic fate [[30], [31], [32], [33]]. A recent report showed, using both Cre and Dre recombinase drivers, that PDGFRα single-positive APs and PDGFRα/PDGFRβ double-positive APs contributed to cold-induced beige adipogenesis whereas PDGFRβ single-positive APs did not, as observed in a mouse model. Moreover, PDGFRα single-positive APs highly expressed *Dpp4* and *Pi16* compared with PDGFRα/PDGFRβ double-positive APs, which highly expressed *Lpl* and *Fabp4* and are committed to an adipogenic fate [34]. However, how these subtypes of APs contribute to adipogenesis under HFD-fed conditions remains unknown.

In this study, we used a PDGFRα-GFP-Cre-ER^T2^/tdTomato (KI/td) mouse model to investigate the role of PDGFRα^+^ APs in HFD-fed male mice. Contrary to other reports, our findings revealed that in male mice, iWAT expanded significantly through *de novo* adipogenesis. We found a higher number of tdTomato ^+^ APs and adipocytes in the iWAT than in the gWAT of male mice. Moreover, tdTomato ^+^ APs showed higher expression levels of *Dpp4* and *Pi16*, and their expression was significantly higher in the iWAT than in the gWAT of HFD-fed male mice.

## 2 Materials and methods

### 2.1 Animals

#### 2.1.1 Generation and housing of KI/td mice

All experimental and animal care procedures were approved by the Animal Experiment Committee of the University of Toyama (Approval number A2018med-142/A2018MED-52). We generated KI/td mice by crossing PDGFRα-GFP-Cre-ER^T2^ knock-in (KI/+) mice [35] with B6.Cg-Gt (ROSA)26Sor^tm9(CAG-td-Tomato)Hze^/J mice [36]. Animals were maintained under a standard 12:12-hour light-dark cycle. Both male and female mice were used for the experiments. The mice had free access to NC (CE-2 CLEA, Japan) or HFD (60% Calories Fat Research Diets, Japan) and water. Body weight (BW) and food intake measurements were made in animals that were housed at 2–3 mice per cage.

### 2.2 Method details

#### 2.2.1 Genotyping

Tail tissue was lysed with the Direct PCR (Tail) lysis solution (Viagen Biotech) and proteinase K (Roche Diagnostics), in accordance with the manufacturer's instructions, to obtain genomic DNA. PCR was performed with crude genomic DNA using the Tks Gflex DNA polymerase kit from TAKARA (Shiga, Japan).

For KI/+ mice, PCR conditions included one cycle at 94 °C for 2 min and 30 cycles at 98 °C for 10 s, 60 °C for 30 s, and 68 °C for 1 min. The following primers were purchased from Invitrogen™ Life Technology (Tokyo, Japan): 5′-WT: AAGACGATTCACACTGCCGATG, 3′-WT: AGACAGCTGAGGACCAGAAAGA, and 3′-KI: TGGTGCAGATGAACTTCAGGGT [35]. DNA fragment sizes were 525 bp for the KI allele and 388 bp for the WT allele. PCR conditions for B6.Cg-Gt (ROSA)26Sor^tm9(CAG-td-Tomato)Hze^/J were as follows: one cycle at 94 °C for 2 min; 10 cycles at 94 °C for 20 s, 65 °C for 15 s (−0.5 °C each cycle), and 68 °C for 10 s; 28 cycles at 94 °C for 15 s, 60 °C for 15 s, and 72 °C for 10 s; one cycle at 72 °C for 2 min; and hold at 10 °C for infinity. The expected DNA fragment size was 200 bp for mutant mice and 297 bp for WT mice. The following primers were purchased from Invitrogen™ Life Technology (Tokyo, Japan): WT FW, AAGGGAGCTGCAGTGGAGTA; WT Rev, CCGAAAATCTGTGGGAAGTC; Mutant FW, CTGTTCCTG0TACGGCATGG; and Mutant Rev, GGCATTAAAGCAGCGTATCC. For KI/+ and tdTomato, PCR products were run on 2% and 1.5% agarose gels (Nippon Gene), respectively, for 30 min. Ethidium bromide (1:1000) was added to visualize DNA on the gel.

#### 2.2.2 Tamoxifen administration

Tamoxifen (TAM; Sigma–Aldrich) was dissolved in sunflower oil (Fujifilm WAKO) and administered to KI/td mice orally at the age of 7 weeks for five consecutive days at 225 mg/kg BW, as described previously [36,37]. After a recovery period of one week, the mice were analyzed for recombination efficiency or fed either NC or HFD. Recombination efficiency was measured using the following formula for immunohistochemistry and FlowJo analysis for flow cytometry:

$$\text{Recombination}\;\text{efficiency}\left(\text{\%}\right)=\frac{{\text{tdTomato}}^{+}\text{cells}}{\text{Total}\;{\text{GFP}}^{+}\text{cells}}\times 100$$

#### 2.2.3 White adipose tissue depot collection

gWAT was clearly identified with forceps along the epididymis and testes in males (also known as epididymal or eWAT) and around the uterus and ovarian ducts in females.

iWAT depot was identified with forceps as extending from the hind limb ventrally to the groin area in both male and female mice, as demonstrated previously [38,39].

#### 2.2.4 Weight of WAT as a percentage of BW

WAT weight as a percentage of BW was calculated using the formula given below:

$$\text{WAT}\;\text{depot}\;\text{weight}\;\text{as}\;\text{\%}\;\text{of}\;\text{BW}=\frac{\text{Depot}\;\text{weight}\left(\text{g}\right)}{\text{Total}\;\text{BW}\left(\text{g}\right)}\times 100$$

#### 2.2.5 Flow-cytometric analysis

The stromal cell suspension was prepared for flow cytometry, as described previously [[40], [41], [42], [43], [44]]. In brief, the tissue was digested in 2 mg/mL collagenase (Sigma) for 45 min at 37 °C and then filtered through a 100 μm cell strainer to collect single cells. For flow cytometry, the 7AAD^−^ population was gated for further analysis of lineage negative (CD31^−^CD45^−^) populations, followed by the positive selection of Sca1^+^PDGFRα^+^tdTomato^+^ populations. Unstained, isotype controls and fluorescence minus one (FMO) were used for justification of the gating strategy. Experiments were performed on the BD FACS Aria™ II machine, and data were analyzed using the FlowJo offline software.

#### 2.2.6 Immunohistochemistry

Hematoxylin and eosin (H&E) staining was performed on 5–10-μm-thick paraffin sections, as described previously [42], and images were taken using an Olympus microscope (Olympus, DP70). Immunohistochemical staining was performed using paraffin-embedded tissue sections, as described previously [42,43,45,46]. Primary and secondary antibodies were used in accordance with the manufacturer's instructions. Primary antibodies including guinea pig anti-GFP (1:200) (Frontier Institute), goat anti-tdTomato (1:150) (SICGEN), rabbit anti-RFP (mouse) (1:250) (Rockland), rabbit anti-Ki67 (1:100) (Abcam), and rabbit anti-perilipin (1:100) (Santa Cruz) antibodies were used. Secondary antibodies and DAPI (Molecular Probes) were used at 1:250 and 1:500 dilutions, respectively. All images were taken using a confocal microscope (Leica TCS-SP5) and the images were analyzed using the ImageJ software.

#### 2.2.7 Percent *de novo* adipogenesis and Ki67-positive cells

Perilipin^+^ (green) adipocytes were considered as pre-existing adipocytes, whereas Perilipin^+^/tdTomato^+^ adipocytes (red or yellow) were considered as newly generated adipocytes. Cell counts to analyze *de novo* adipogenesis were determined through multi-point counting using the ImageJ software (confocal images included 5 randomly selected areas from one depot per slide per specimen, with 4–6 specimens under each condition). The percentages of proliferative APs (Ki67^+^/tdTomato^+^ cells) and newly generated adipocytes were calculated using the following formulae, respectively.

$$\text{\%}\;\text{of}\;\text{Ki}67^{+}/{\text{tdTomato}}^{+}\text{cells}=\frac{\text{Ki}67^{+}/{\text{tdTomato}}^{+}\text{cells}}{\text{Total}\;\text{no.}\;\text{of}\;\text{DAPI}}\times 100$$

$$\%\;\text{of}\;\text{newly}\;\text{generated}\;\text{adipocytes}=\frac{{\text{tdTomato}}^{+}/{\text{perilipin}}^{+}\text{adipocytes}}{\text{Total}\;{\text{tdTomato}}^{-}/{\text{perilipin}}^{+}\text{adipocytes}}\times 100$$

#### 2.2.8 EdU staining

We injected EdU into 12-week HFD-fed KI/td male mice at 2 mg/kg BW. Three hours later, the mice were euthanized to harvest the tissues. Frozen sections of adipose tissue (35–40 μm thickness) were prepared, as described previously [42]. EdU immunostaining was performed using Alexa 555 and anti-RFP antibodies, followed by the secondary antibody. The percentage of EdU^+^ APs was calculated using the formula given below:

$$\%\;\text{of}\;{\text{EdU}}^{+}/{\text{tdTomato}}^{+}\text{cells}=\frac{{\text{EdU}}^{+}/{\text{tdTomato}}^{+}\text{cells}}{\text{Total}\;\text{no.}\;\text{of}\;\text{DAPI}}\times 100$$

#### 2.2.9 Adipocyte size measurement

The mean adipocyte number was counted using the multi-point tool in ImageJ 1.53a (National Institute of Health, USA). The adipocyte size was measured using the “Set Scale” function in ImageJ 1.53a from five random 20 × fields per specimen. We took one depot per slide per specimen and 4–6 specimens for each condition.

#### 2.2.10 Real-time polymerase chain reaction

FACS-sorted cells were collected in RPMI buffer and stored in Isogen (Nippon Gene). The Qiagen RNeasy kit was used to extract RNA from the cells. The TaKaRa PrimeScript RNA Kit was used for reverse transcription, according to the manufacturer's instructions. The quantitative PCR amplification reaction was performed using gene-specific primers and TB Green™ Premix Taq™ Ⅱ (Tli RNaseH Plus; Takara, Shiga Japan), as per the manufacturer's instructions, and normalized to the expression level of *Tf2b*.

### 2.3 Statistical analysis

An unpaired Student's *t*-test was used for statistical analyses. The results are expressed as mean ± SEM, and ∗*p* < 0.05 and ∗∗*p* < 0.01 were considered as being indicative of statistically significant differences.

## 3 Results

### 3.1 Generation of smaller adipocytes in the iWAT of HFD-fed male mice

**Figure 1:**
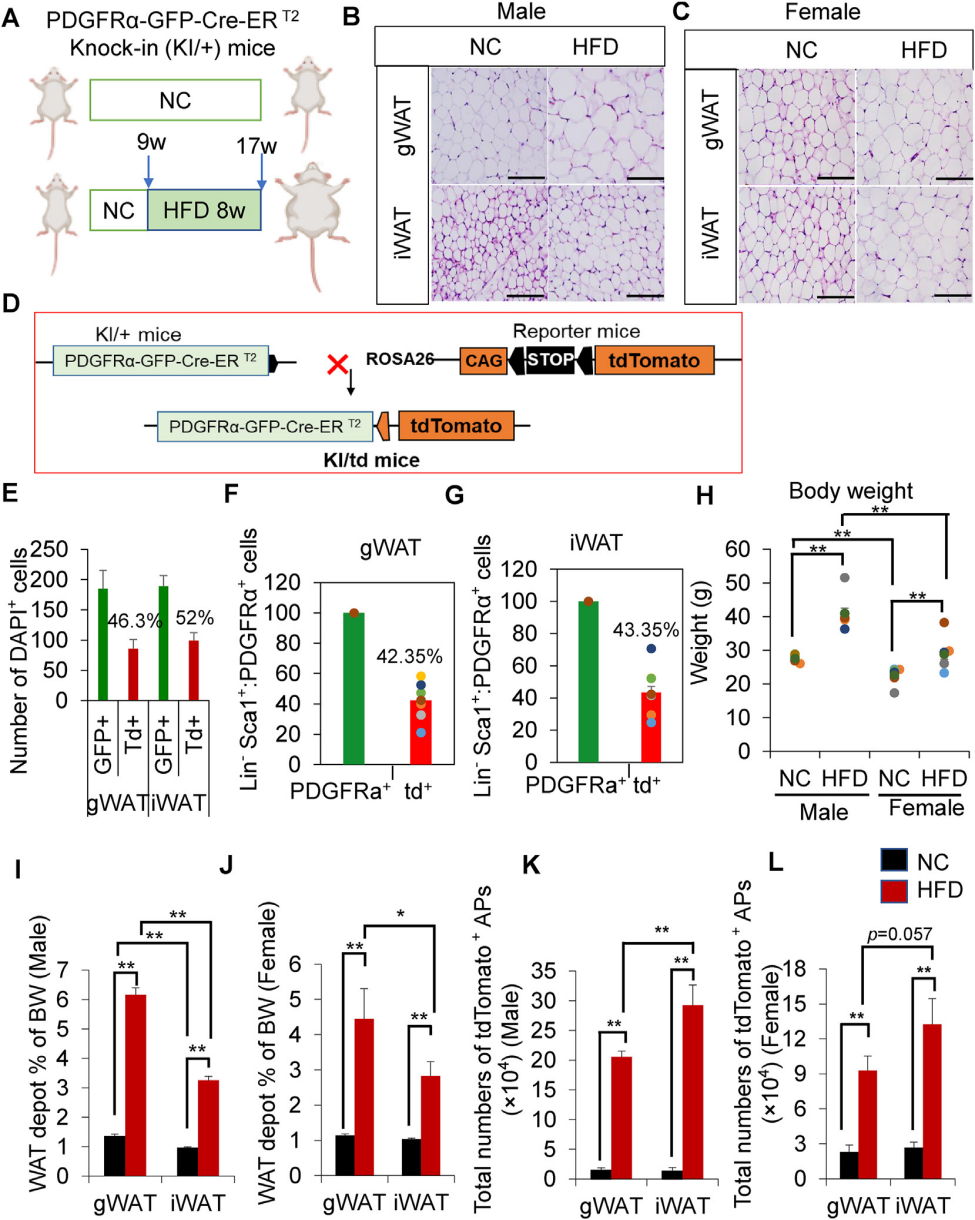
**Generation of smaller adipocytes in the iWAT of HFD-fed male mice. (A)** Schematic diagram of the experimental protocol. **(B)** Representative images of hematoxylin and eosin (H&E)-stained paraffin sections of gWAT and iWAT obtained from KI/+ male mice fed either NC or HFD for 8 weeks. Mice were started on HFD at 9 weeks of age and euthanized at 17 weeks. Scale bar = 250 μm (*n* = 4/group). **(C)** Representative images of H&E-stained paraffin sections of gWAT and iWAT obtained from KI/+ female mice fed either NC or HFD for 8 weeks. Mice were started on HFD at 9 weeks of age and euthanized at 17 weeks. Scale bar = 250 μm (*n* = 3/group). **(D)** Graphical diagram for the generation of KI/td mice. **(E**–**G)** Representative graphs of recombination efficiency analyzed by immunohistochemistry **(E)** and flow cytometry analysis in both depots **(F and G).** Tamoxifen (TAM) was administered at the age of 7 weeks for five consecutive days. Mice were euthanized at 9 weeks of age; gWAT and iWAT were collected and subjected to immunohistochemistry and FACS analysis (*n* = 8/group). **(H)** Body weight (g) of male and female KI/td mice fed either NC or HFD for 8 weeks. Mice were started on HFD at 9 weeks of age and euthanized at 17 weeks (Male: NC, *n* = 8; HFD, *n* = 8; female: NC, *n* = 8; HFD, *n* = 8). **(I and J)** Weight of the WAT depots (gWAT and iWAT) as % BW in male **(I)** and female **(J)** KI/td mice fed either NC or HFD for 8 weeks (Male: NC, *n* = 8; HFD, *n* = 8; female: NC, *n* = 8; HFD, *n* = 8) (BW; body weight). **(K and L)** Total number of tdTomato^+^ APs (CD31^−^CD45^−^Sca1^+^ cells) determined by flow cytometry in male **(K)** and female **(L)** KI/td mice fed either NC or HFD for 8 weeks. (Male: NC, *n* = 8; HFD, *n* = 7; female: NC, *n* = 5; HFD, *n* = 6). Data represent mean ± SEM. Statistical analysis was performed using Student's *t*-test (∗*p* < 0.05, ∗∗*p* < 0.01).

To investigate the effect of HFD on the proliferative potential and differentiation capacity of PDGFRα^+^ APs, we used a PDGFRα-GFP-Cre-ER^T2^ knock-in (KI/+) mouse model (Figure 1A). Both male and female HFD-fed mice showed similar BW gain and a significant increase in weight in both gWAT and iWAT compared with control NC-fed mice (Supplementary Figs. S1A–S1C). We found that HFD-fed mice consumed lower amounts of food than NC-fed mice, although their caloric intake was comparable (Supplementary Fig. S2A). Overall, our findings revealed that caloric intake from HFD significantly increased BW and depot weight in both male and female mice. Our histological analysis revealed an increased number of smaller adipocytes in iWAT compared with that in gWAT in male mice fed NC or HFD (Figure 1B); however, no such difference was observed in female mice (Figure 1C). Consistent with histological data, the number of larger adipocytes was higher in the gWAT than in the iWAT of male mice; however, an almost similar pattern was observed in the depots of HFD-fed female mice (Supplementary Fig. S2B). We hypothesize that the APs in the iWAT of HFD-fed male mice showed a greater proliferative and differentiation potential than the APs in the gWAT of HFD-fed male mice.

### 3.2 Newly formed APs proliferate in the iWAT of HFD-fed male mice

To investigate the role of PDGFRα^+^ APs in HFD-induced adipogenesis, we generated genetically engineered KI/td mice by crossing KI/+ mice with Rosa26-td-Tomato mice (Figure 1D). We administered TAM orally at 225 mg/kg BW for 5 consecutive days (Supplementary Fig. S3A) and found that TAM administration did not alter the BW (Supplementary Fig. S3B). Then, we confirmed the recombination efficiency (46.3% in gWAT and 52% in iWAT) using immunohistochemistry (% was calculated using the formula given in the Methods section) (Figure 1E). We also confirmed the recombination efficiency by flow cytometry analysis (42.35% in gWAT and 43.35% in iWAT) (Figure 1F,G).

To investigate the effect of HFD on the number of PDGFRα^+^ APs, we fed KI/td mice either HFD or NC for 8 weeks after TAM administration. HFD feeding significantly increased the fat depot percentage relative to BW, regardless of sex, compared with NC feeding (Figure 1H–J). Furthermore, iWAT restrained weight gain compared with gWAT (Figure 1I,J).

Next, we performed flow cytometry analysis to evaluate the number of lineage-negative (CD31^−^CD45^−^), Sca1^+^PDGFRα^+^, and tdTomato^+^ (tdTomato^+^ APs) populations. The gating strategy is shown in Supplementary Figs. S3C and S3D. The number of tdTomato^+^ APs significantly increased in the gWAT and iWAT of HFD-fed mice, regardless of their sex (Figure 1K, L and Supplementary Figs. S4A and S4B). These data also indicate that in HFD-fed male mice, the iWAT has a higher number of tdTomato^+^ APs than the gWAT (Figure 1K), suggesting that HFD contributes to the increase in tdTomato^+^ APs in iWAT.

**Figure 2:**
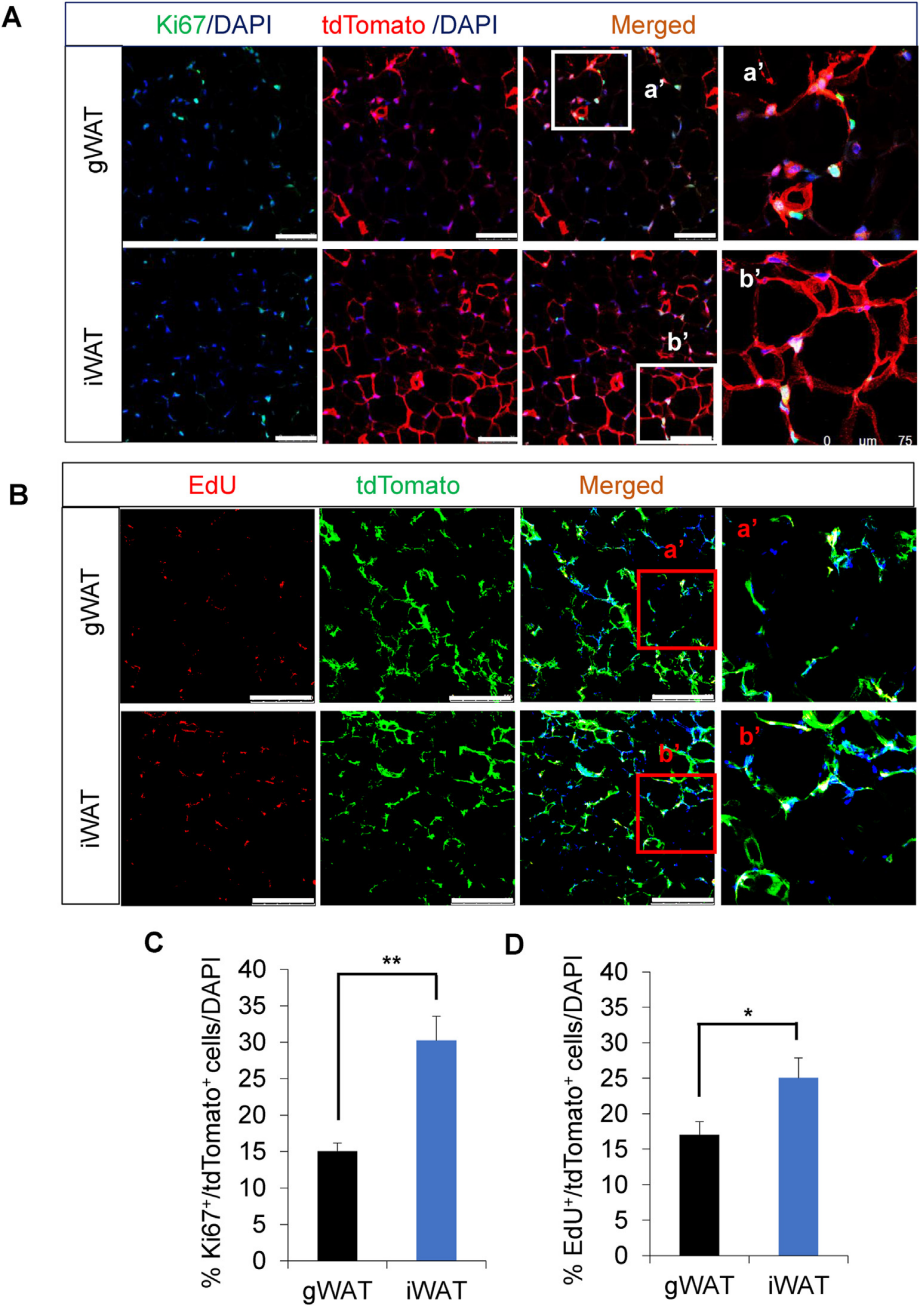
**Proliferative potential of newly formed APs in the iWAT of male mice upon HFD feeding. (A)** Representative confocal images of gWAT and iWAT stained with anti-Ki67 (green) and anti-tdTomato (red) antibodies in KI/td male mice fed HFD for 8 weeks. Mice were started on HFD at 9 weeks of age and euthanized at 17 weeks (scale bar = 75 μm; *n* = 5). **(B)** Representative confocal images of gWAT and iWAT frozen sections stained with EdU (red) and anti-tdTomato (green) antibodies in HFD-fed KI/td male mice (scale bar = 250 μm; *n* = 3). **(C)** Quantification of Ki67^+^/tdTomato^+^ cells/DAPI cells in both depots of KI/td male mice fed HFD for 8 weeks (*n* = 5). **(D)** Quantification of EdU^+^/tdTomato^+^ cells/DAPI cells in both depots of HFD-fed KI/td male mice (*n* = 3). Data represent mean ± SEM. Statistical analysis was performed using Student's *t*-test; (∗*p* < 0.05, ∗∗*p* < 0.01).

To further investigate the proliferative potential of newly formed tdTomato^+^ APs, we stained the paraffin sections of WAT with anti-Ki67 and anti-tdTomato antibodies and found that the percentage of Ki67^+^/tdTomato^+^ double-positive cells was significantly higher in iWAT than in gWAT of both male and female mice fed HFD for 8 weeks (Figure 2A,C, and Supplementary Figs. S5A and S5B). Next, we injected HFD-fed KI/td male mice with EdU to trace its cellular uptake and found that EdU uptake was significantly increased in the tdTomato^+^ cells of iWAT compared with that of gWAT (Figure 2B,D), indicating that newly formed tdTomato^+^ APs proliferate in response to HFD. Overall, our results suggest that in male mice, APs in iWAT have more proliferative capacity than those in gWAT.

### 3.3 *de novo* adipogenesis in the iWAT of HFD-fed male mice

**Figure 3:**
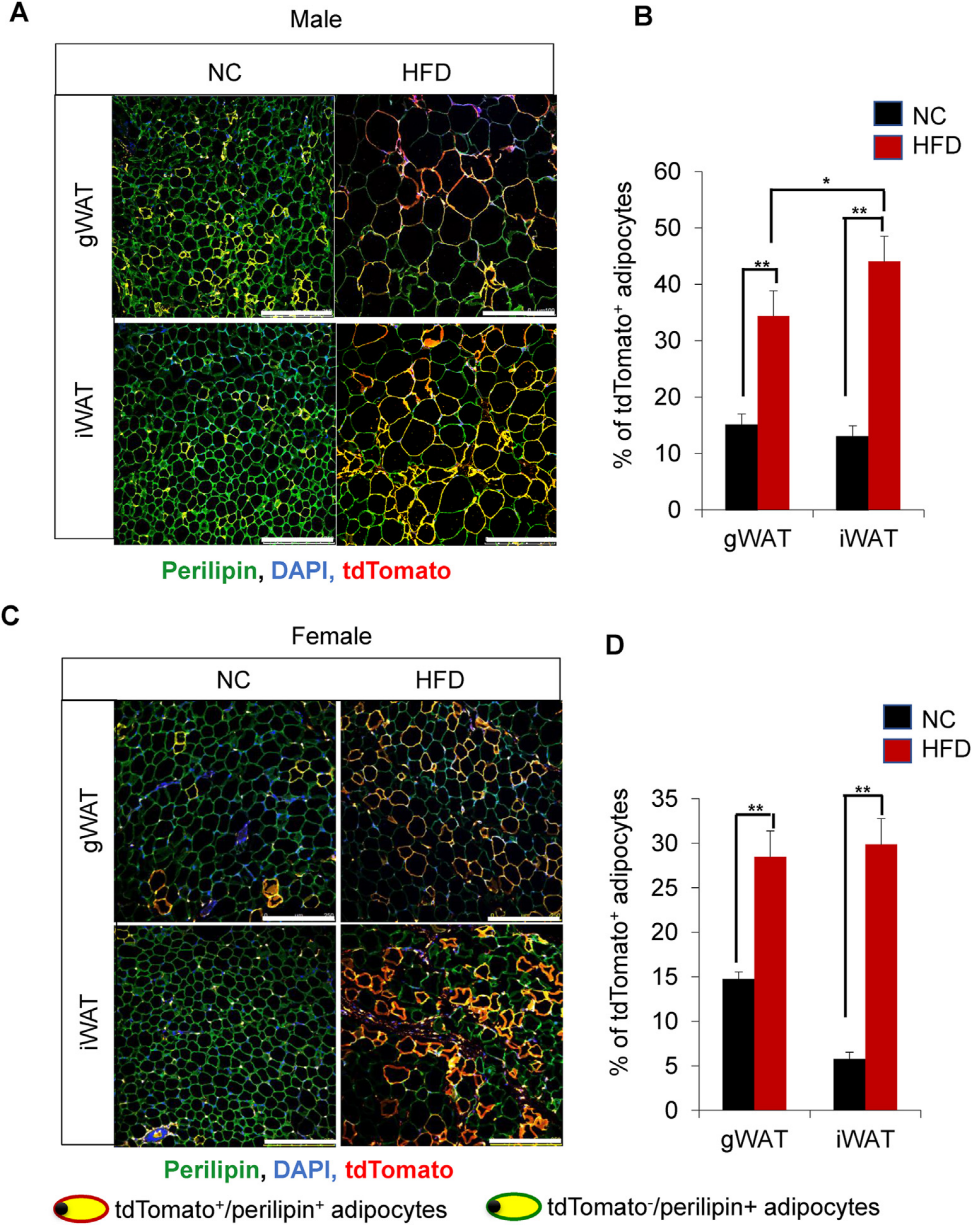
***De novo* adipogenesis in the iWAT of HFD-fed male mice. (A)** Representative confocal images of gWAT and iWAT stained with anti-perilipin (green) and anti-tdTomato (red) antibodies in KI/td male mice fed either NC or HFD for 8 weeks. Mice were started on HFD at 9 weeks of age and euthanized at 17 weeks (scale bar = 250 μm). **(B)** Quantification of tdTomato^+^ adipocytes in the gWAT and iWAT of KI/td male mice (NC, *n* = 6; HFD, *n* = 10). **(C)** Representative confocal images of gWAT and iWAT stained with anti-perilipin (green) and anti-tdTomato (red) antibodies in KI/td female mice fed either NC or HFD for 8 weeks (scale bar = 250 μm). **(D)** Quantification of tdTomato^+^ adipocytes in the gWAT and iWAT of KI/td female mice (NC, *n* = 6; HFD, *n* = 9). Data represent mean ± SEM. Statistical analysis was performed using Student's *t*-test (∗*p* < 0.05, ∗∗*p* < 0.01).

**Figure 4:**
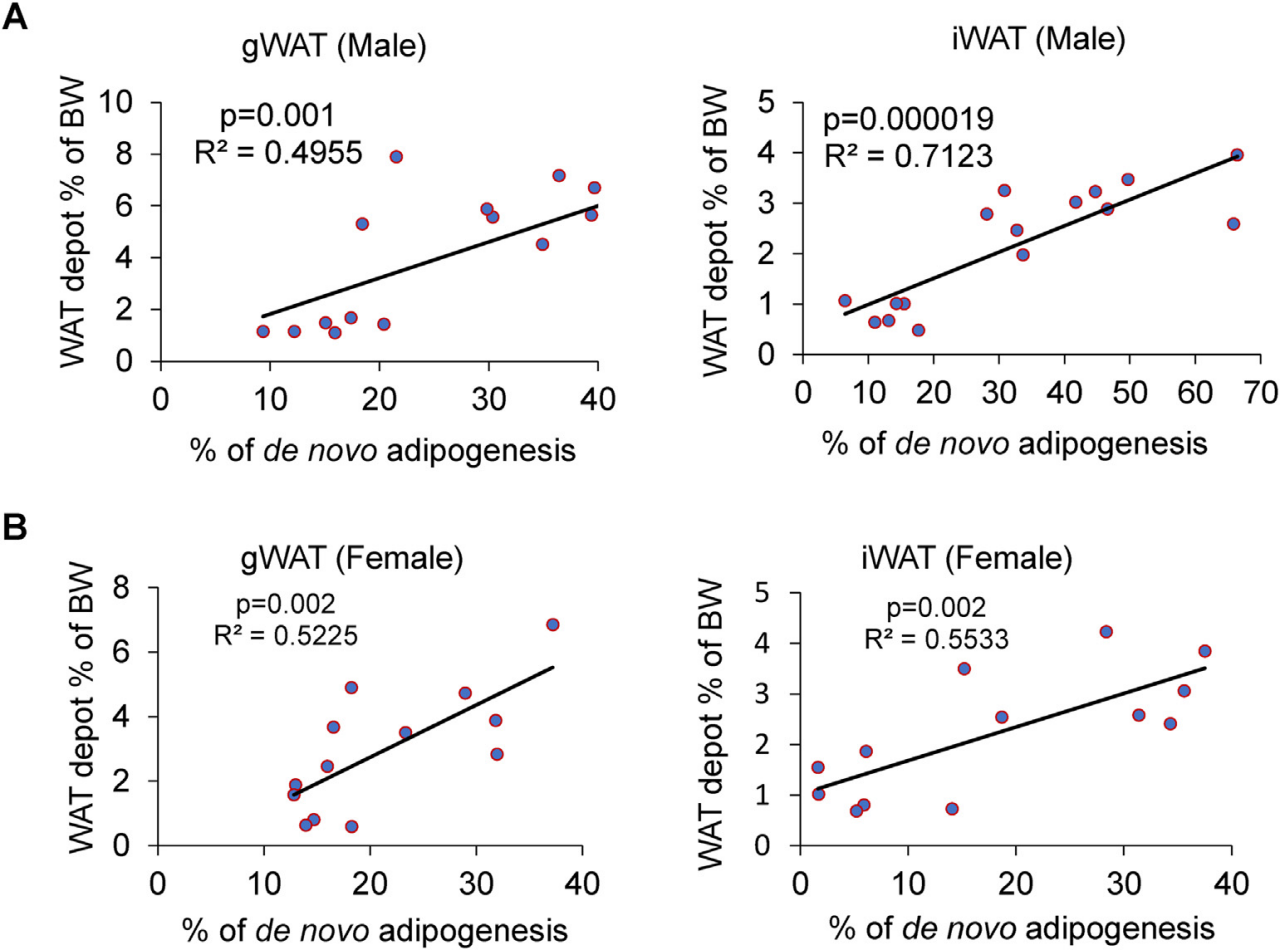
***De novo* adipogenesis contributes more to iWAT expansion in HFD-fed male mice. (A)** Correlation between the weights of the WAT depots (gWAT and iWAT) as % BW and % *de novo* adipogenesis (hyperplasia) in KI/td male mice fed either NC or HFD for 8 weeks. Mice were started on HFD at 9 weeks of age and euthanized at 17 weeks. Each point indicates an individual mouse (NC, *n* = 6; HFD, *n* = 10). **(B)** Correlation between the weights of the WAT depots (gWAT and iWAT) as % BW and % *de novo* adipogenesis (hyperplasia) in KI/td female mice fed either NC or HFD for 8 weeks. Mice were started on HFD at 9 weeks of age and euthanized at 17 weeks. Each point indicates an individual mouse (NC, *n* = 6; HFD, *n* = 7).

To investigate *de novo* adipogenesis in HFD-fed mice, we stained paraffin sections of WAT with anti-perilipin and anti-tdTomato antibodies (Figure 3). We found that tdTomato^+^ APs significantly differentiated into tdTomato-labeled adipocytes in the gWAT and iWAT of HFD-fed mice, irrespective of the sex of the animals (Figure 3A,C, and Supplementary Figs. S6A and S6B). Upon HFD feeding, the percentage of newly generated tdTomato^+^/perilipin^+^ adipocytes increased in male mice (from 15.1% to 34.4% in gWAT and from 13.0% to 44.1% in iWAT) (Figure 3B). In female mice, the percentage of tdTomato^+^ adipocytes increased from 14.8% to 28.5% in gWAT and from 5.8% to 29.9% in iWAT (Figure 3D). Next, to investigate the contribution of hyperplasia in WAT expansion, we performed correlation-comparison analysis between % *de novo* adipogenesis and WAT depot as % BW. In male mice, we found that the correlation was stronger in the iWAT than in the gWAT (Figure 4A); however, no such difference was observed in female mice (Figure 4B). Our data clearly demonstrate that *de novo* adipogenesis contributes more to iWAT expansion than to gWAT expansion, suggesting that iWAT expands through hyperplasia in response to HFD stimuli.

### 3.4 tdTomato^+^APs in iWAT underwent hyperplasia and tdTomato^+^APs in gWAT underwent hypertrophy in HFD-fed male mice

**Figure 5:**
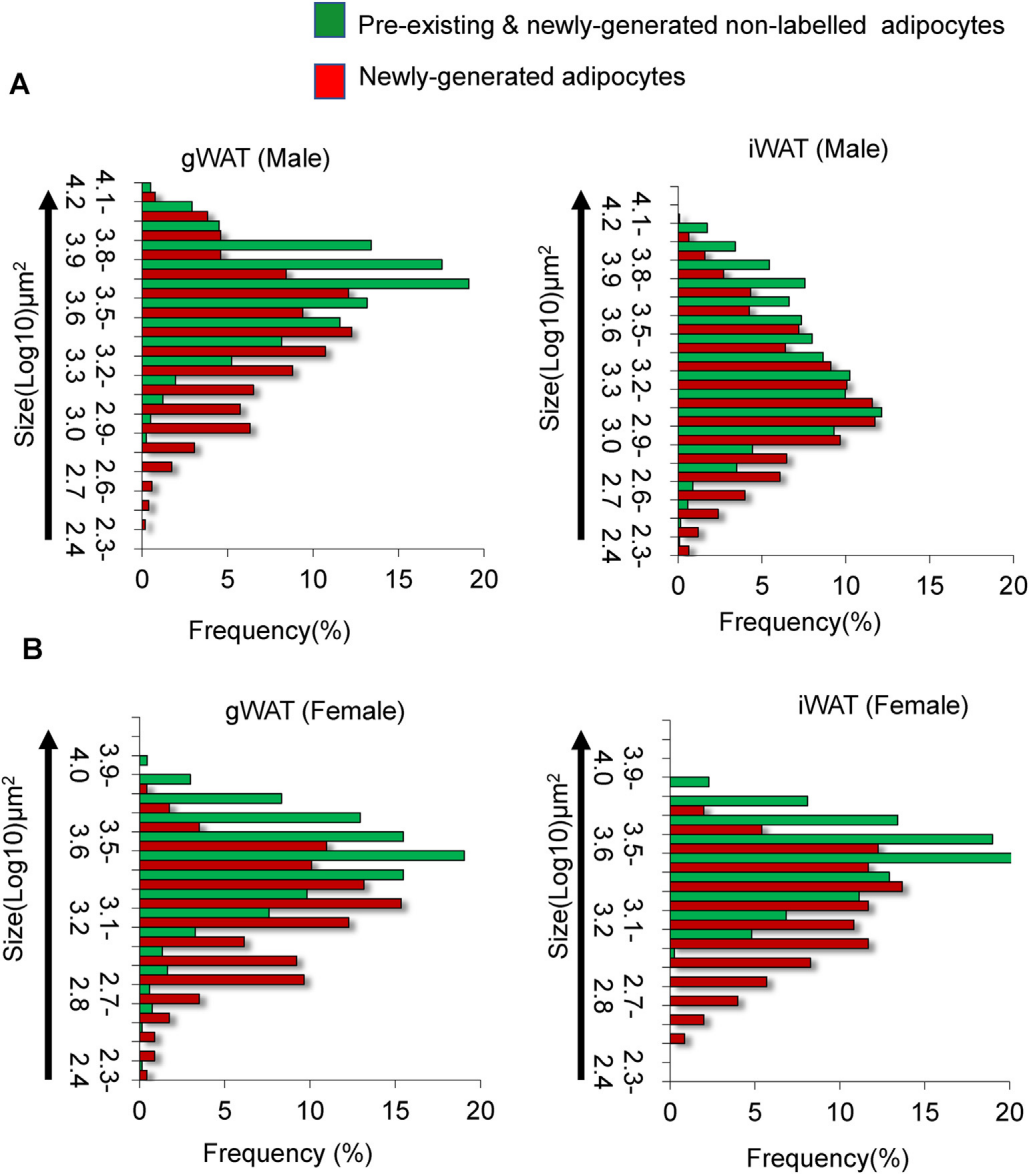
**HFD accelerates the recruitment of smaller adipocytes in the iWAT of male mice. (A)** Frequency distribution of adipocyte size (area μm^2^) in KI/td male mice fed HFD for 8 weeks. Mice were started on HFD at 9 weeks of age and euthanized at 17 weeks. The red bar (tdTomato^+^) represents newly generated adipocytes, whereas green (tdTomato^−^) represents a mixture of pre-existing and newly generated non-labeled adipocytes (*n* = 5). **(B)** Frequency distribution of adipocyte size (area μm^2^) in KI/td female mice fed HFD for 8 weeks. The red bar (tdTomato^+^) represents newly generated adipocytes, whereas green (tdTomato^−^) represents a mixture of pre-existing and newly generated non-labeled adipocytes (*n* = 4).

We analyzed the size of the pre-existing adipocytes and newly generated adipocytes in both fat depots (Figure 3A,C). We considered cells with tdTomato^+^/perilipin^+^ co-immunofluorescence as newly generated tdTomato^+^ adipocytes. tdTomato^−^/perilipin^+^ adipocytes consisted of pre-existing adipocytes as well as newly generated tdTomato^−^ adipocytes derived from non-recombined APs (Supplementary Fig. S7). Regarding adipose depots in male mice, adipocyte size frequency histogram data showed that HFD increases the generation of smaller adipocytes in iWAT and that of larger adipocytes in gWAT (Figure 5A). In contrast, almost similar patterns were observed in the two fat depots in female mice (Figure 5B). Gene expression analysis further revealed the downregulated expression of inflammatory marker genes in iWAT compared with that observed in gWAT (Supplementary Fig. S8), suggesting that iWAT is less inflammatory. These data support our hypothesis that in male mice, iWAT undergoes hyperplasia due to the recruitment of newly generated adipocytes, whereas gWAT mainly undergoes hypertrophy through the enlargement of the pre-existing adipocytes. Interestingly, the size of the tdTomato^−^ adipocyte, which may contain newly generated adipocytes (derived from non-labeled APs), was larger than that of the tdTomato^+^ adipocyte.

**Figure 6:**
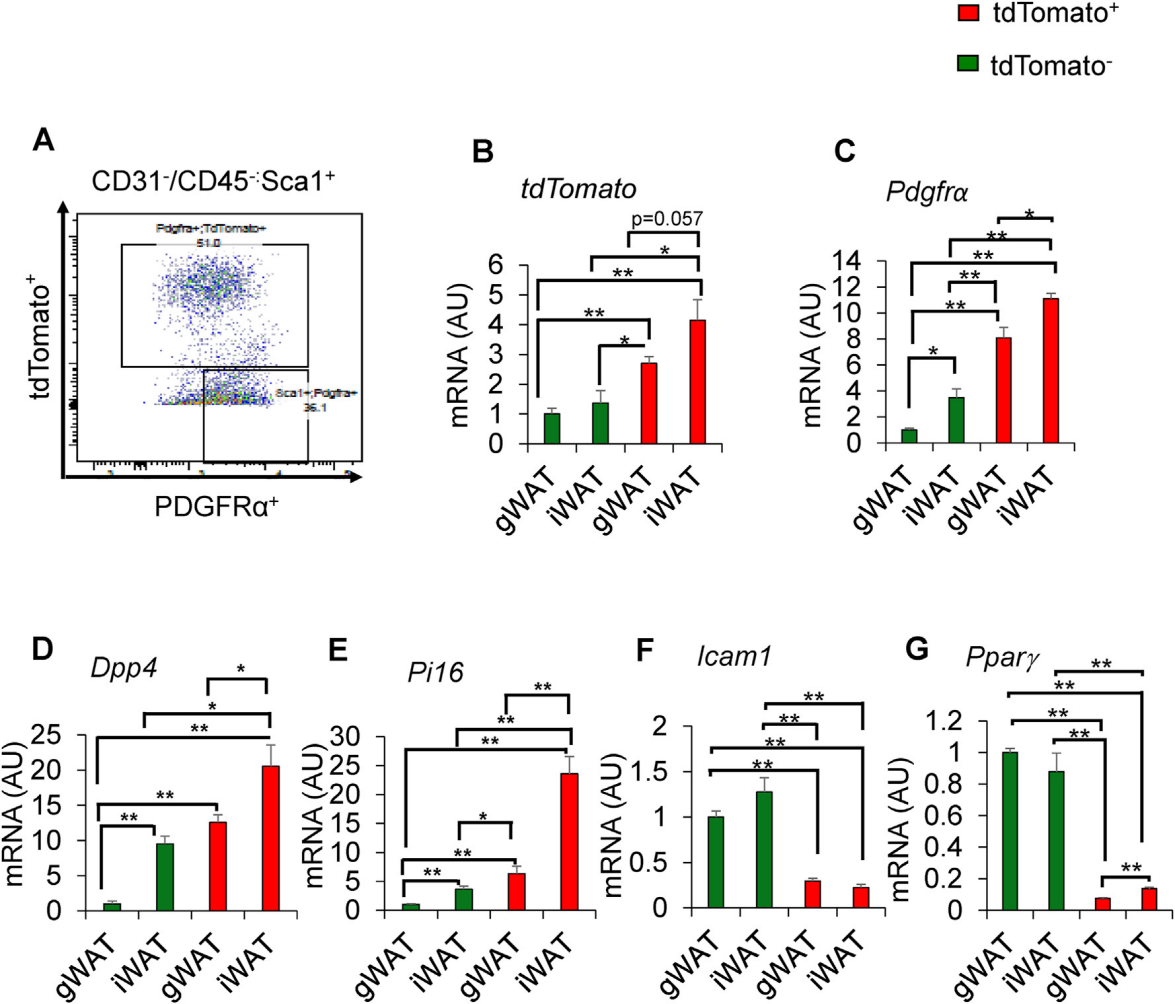
**Gene expression analysis of FACS-sorted tdTomato**^**+**^**labeled and non-labeled APs. (A)** Representative flow cytometry image showing the gating strategy for isolating tdTomato^+^ and tdTomato^−^ APs from KI/td male mice fed HFD for 8 weeks. Mice were started on HFD at 9 weeks of age and euthanized at 17 weeks. **(B–G)** Gene expression analysis of markers of adipocyte progenitors in FACS-isolated fractions of tdTomato^+^ and tdTomato^−^ APs from KI/td male mice fed HFD for 8 weeks (*n* = 3). Data represent mean ± SEM. Statistical analysis was performed using Student's *t*-test (∗*p* < 0.05, ∗∗*p* < 0.01).

To investigate the intrinsic differences between the APs in the two WAT depots in HFD-fed male mice, we sorted tdTomato^+^ and tdTomato^−^ APs using fluorescence-activated cell sorting (FACS) (Figure 6A) and the sorted cells were subjected to gene expression analysis. Isolated tdTomato ^+^ APs showed higher mRNA levels of *tdTomato* and *Pdgfrα* (Figure 6B,C)*.* Interestingly, tdTomato ^+^ APs showed higher expression levels of *Dpp4* and *Pi16* than tdTomato^−^ APs, and the expression of these genes was significantly higher in the iWAT than in the gWAT of male mice fed HFD for 8 weeks (Figure 6D,E). As we have already mentioned that iWAT possesses large numbers of tdToamao^+^ cells, taken together these data suggest that the APs in iWAT are highly proliferative in response to HFD stimuli. By contrast, tdTomato^−^ APs showed higher expression levels of *Icam1* and *Pparγ*, suggesting that tdTomato^−^ APs are more committed to an adipogenic fate (Figure 6F,G).

## 4 Discussion

The ability of APs to proliferate and undergo adipogenesis during obesity is important to maintain a healthy expansion of WAT. A previous report showed that only APs in the iWAT had proliferative potential in HFD-fed mice [25]. Fate-mapping studies utilizing different inducible Cre-driver mice have shown that the APs in the iWAT of HFD-fed male mice have little or no proliferative potential [5,6,[26], [27], [28]]. The discrepant results can be explained by the different experimental models of transgenic mice used in different studies. Wang et al. used AdipoChaser mice [27] and Jeffery et al. used Adiponectin-creER; mTmG mice [6,28] to show that HFD-induced adipogenesis occurs only in gWAT. Another report used PDGFRα-MerCreMer-Rosa26-tdTomato mice [26] and showed greater labeling efficiency in gWAT than in iWAT, supporting the hypothesis that only the gWAT in male mice can differentiate. Vishvanath et al. used PDGFRβ^rtTA^;TRE-Cre; mTmG mice [5] and found that 4 weeks of HFD induces adipogenesis in gWAT but not in iWAT. They also showed that the number of APs increased in both fat depots (gWAT and iWAT). However, whether PDGFRβ^+^ APs expressed PDGFRα was not discussed. Gao et al. also used PDGFRβ-Cre mice and demonstrated that PDGFRβ^+^ APs derived from PDGFRα^+^ cells in iWAT contribute to HFD-induced adipogenesis [19]. Han et al. provided key evidence that PDGFRα single-positive and PDGFRα/PDGFRβ double-positive APs, but not PDGFRβ single-positive APs, contribute to cold-induced adipogenesis [34]. Although the number of PDGFRα/PDGFRβ double-positive cells was less than that of PDGFRα single-positive and PDGFRβ single-positive cells, the adipogenic potential of PDGFRα/PDGFRβ double-positive cells was comparable with that of PDGFRα single-positive APs, showing that PDGFRα/PDGFRβ double-positive cells have higher potential for beige adipogenesis. However, they did not analyze how these APs contributed to HFD-induced adipogenesis. Another group used PDGFRα-Cre-ER^T2^ mice generated by Rivers et al. [47] and showed that PDGFRα^+^ APs did not participate in adult adipogenesis [48]. These studies were performed in different laboratories using different doses of TAM for recombination and with distinct Cre-drivers for producing transgenic models. This prompted us to examine the proliferative and adipogenic capacity of iWAT APs utilizing PDGFRα-GFP-Cre-ER^T2^ knock-in (KI/+) mice. Our knock-in Cre-driver mouse model showed higher labeling efficiency of APs in both iWAT and gWAT than the mouse models reported by River et al. and Ding et al. [47,49]. However, the precise reason for discrepant labeling efficiency of APs within the WAT depots remains unknown.

Taking advantage of single-cell RNA sequence analysis, studies have revealed that DPP4^+^ and Pi16^+^ APs have a high proliferative potential [[30], [31], [32], [33]]. Han et al. reported that PDGFRα single-positive APs showed high expression of *Dpp4* and *Pi16* and contributed to cold-induced adipogenesis. Moreover, PDGFRα/PDGFRβ double-positive APs contributed to cold-induced adipogenesis to a similar extent, although their number was smaller. However, PDGFRα/PDGFRβ double-positive APs showed an upregulation of *Lpl* and *Fabp4* expression, which indicates that these APs are more committed to adipogenesis [34]. Consistent with this, our gene expression analysis revealed higher expression of *Pdgfrα, Dpp4*, and *Pi16* in FACS-purified tdTomato^+^ APs than in non-labeled (tdTomato^−^) APs, whereas tdTomato^−^ (non-recombined) APs showed higher expression of *Icam1* and *Ppar*γ. Collectively, these results indicate that iWAT has an abundance of a subtype of APs with higher expression of *Pdgfr*α*, Dpp4,* and *Pi16* and a higher proliferative potential. Thus, compared with gWAT expansion, iWAT expansion occurs through hyperplasia.

Previously, we and others showed that the generation of smaller adipocytes contributed to insulin sensitivity [[40], [41], [42],[50], [51], [52], [53]]. Adipocyte size has been reported to be directly related to the secretory profile as well as adipocyte functions [25,54]. The average size of newly formed and pre-existing adipocytes within both the WAT depots in mice fed HFD for 8 weeks is comparable in both sexes [6]. According to another study, newly formed adipocytes from PDGFRβ^+^ APs were larger in size than pre-existing adipocytes [19]; in contrast, we found that newly generated adipocytes were smaller in size compared with pre-existing adipocytes. Our data also showed that iWAT had a higher percentage of smaller, newly generated adipocytes than gWAT in male mice fed HFD for 8 weeks.

Our study has some limitations; the recombination efficiency is about 42–43% and tdTomato^−^ non-labeled adipocytes consisted of both pre-existing adipocytes and adipocytes derived from non-recombined APs, which have lower *Pdgfrα* expression. However, we believe that our argument is strong with key evidence that iWAT is rich in APs with higher proliferative and adipogenic capacity, thus resulting in hyperplasia under HFD conditions.

In conclusion, our findings reveal that iWAT APs of male HFD-fed mice proliferate and differentiate into newly generated, smaller adipocytes to a greater extent than that observed in gWAT APs. We believe that iWAT is a potential target for the development of drugs for treating obesity-induced insulin resistance.

## Author contributions

M.B. and A.N. designed and executed the experiments. K.T., M.R.A., and Y.I. helped in performing the *in vivo* experiments. A.N., Y.W., T.K., and J.L. assisted in genotyping and reviewing the manuscript. S.F., J.I., T.N., and K.Y. critically reviewed and revised the manuscript. K.I., M.H., and T.E. provided the founder mice. K.T. conceived the idea of the study and supervised the project. M.B. and A.N. contributed to this study equally.
