# Supplementary material for: Fate of adipocyte progenitors during adipogenesis in mice fed a high-fat diet
Source: Mol Metab. 2021 Sep 23;54:101328. doi: 10.1016/j.molmet.2021.101328 (PMC8495176; doi:10.1016/j.molmet.2021.101328)
Supplement: Supplementary file 1 — Multimedia component 1 [file mmc1.pdf]

Fate of adipocyte progenitors during adipogenesis in mice upon high-fat diet feeding

Muhammad Bilal[1], Allah Nawaz[1,2*], Tomonobu Kado[1], Muhammad Rahil Aslam[1], Yoshiko Igarashi[1], Ayumi Nishimura[1], Yoshiyuki Watanabe[1], Takahide Kuwano[1], Jianhui Liu[1], Hiroyuki Miwa[3], Takumi Era[3], Koichi Ikuta[4], Johji Imura[5], Kunimasa Yagi[1], Takashi Nakagawa[2], Shiho Fujisaka[1], Kazuyuki Tobe[1*].

Supplementary Figures 1-8

Supplementary Figure 1

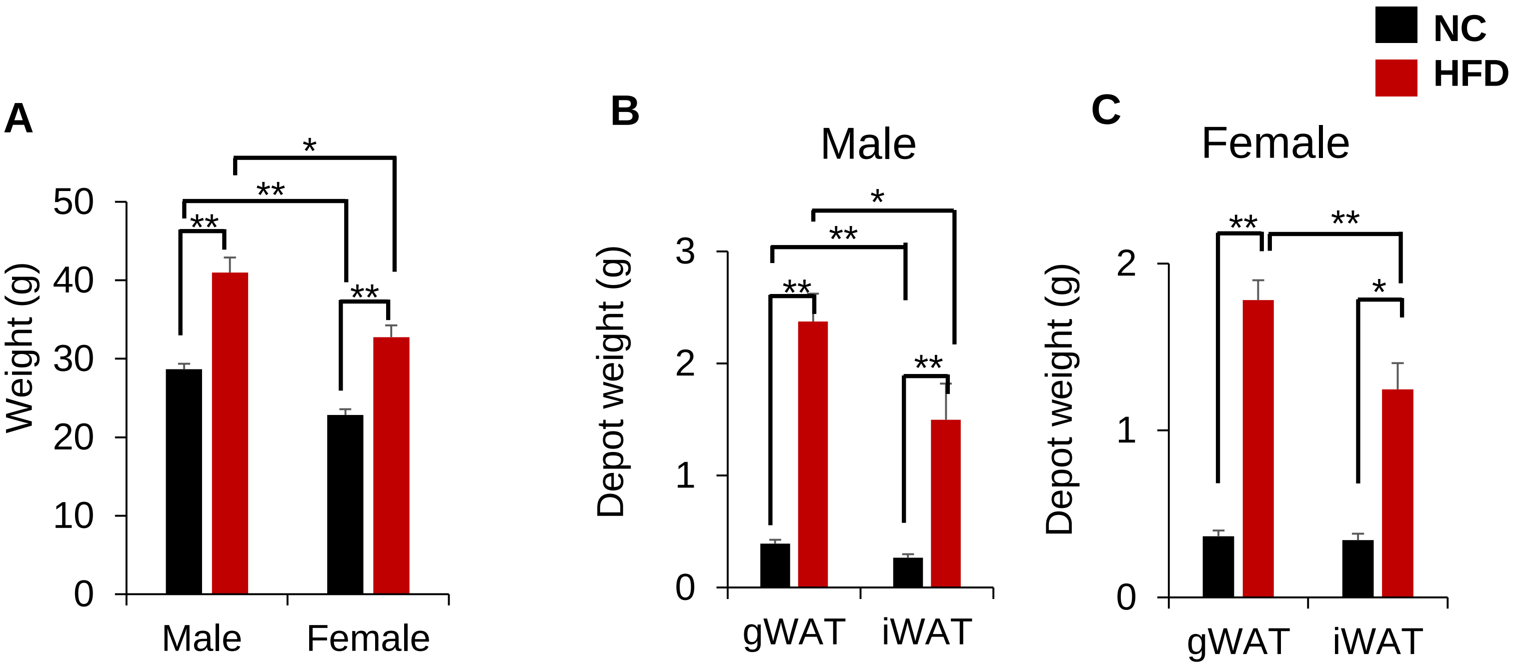

Supplementary Figure 1. Body weight and depot weight of HFD-fed mice. (A) Body weight (g) of KI/+ male and female mice during 8 weeks of HFD-feeding compared with their littermate control NC-fed mice. Mice were started on HFD at 9 weeks of age and euthanized at 17 weeks; n = 8 per group. **(B, C)** Fat depot weight (g) of KI/+ male and female mice during 8 weeks of HFD-feeding compared with their littermate control NC-fed mice. Mice were started on HFD at 9 weeks of age and euthanized at 17 weeks; n = 8 per group. Data represent mean ± SEM. Statistical analysis was performed using Student's *t*-test (*$p < 0.05$, **$p < 0.01$).

Supplementary Figure 2

A

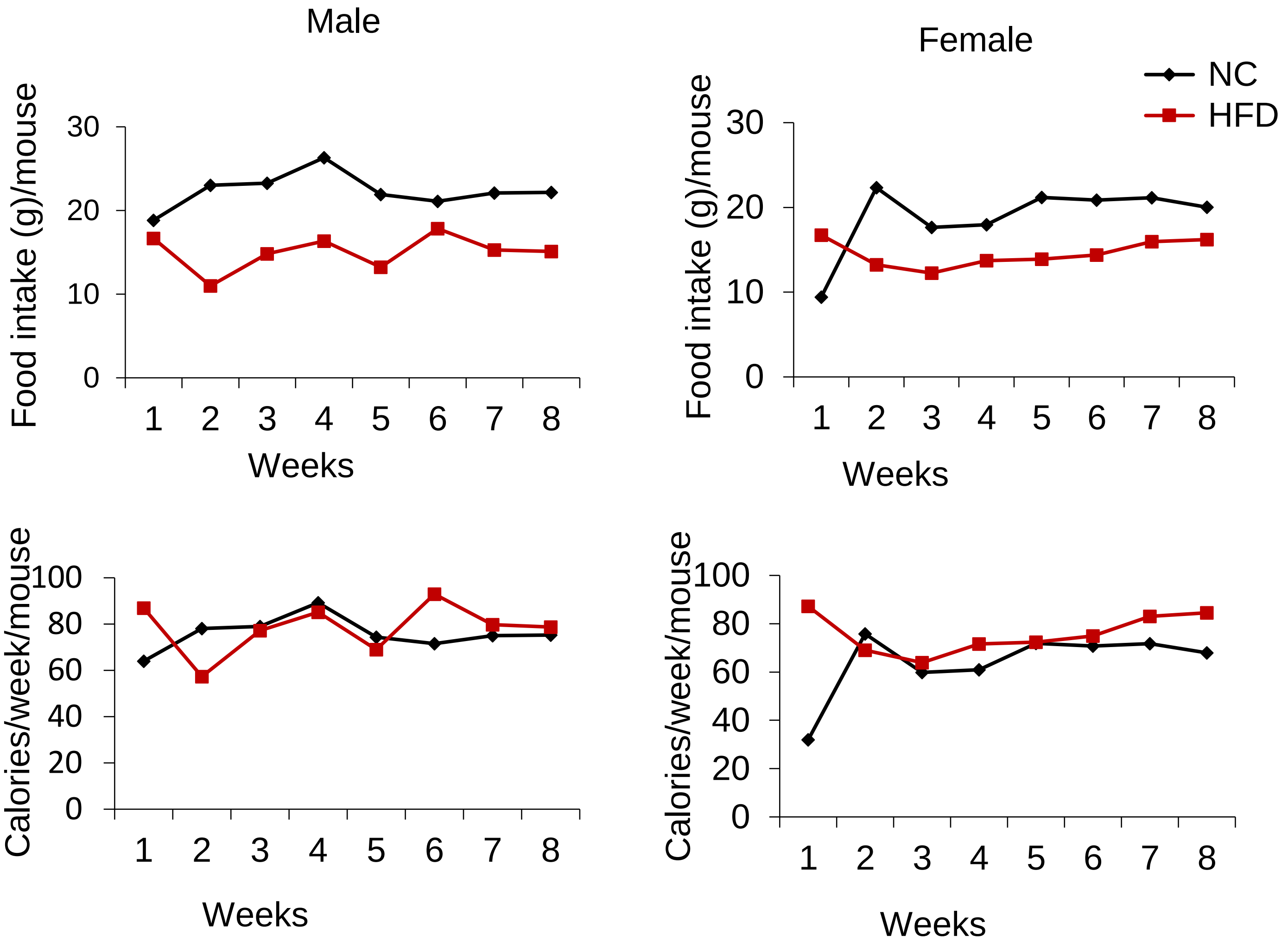

B

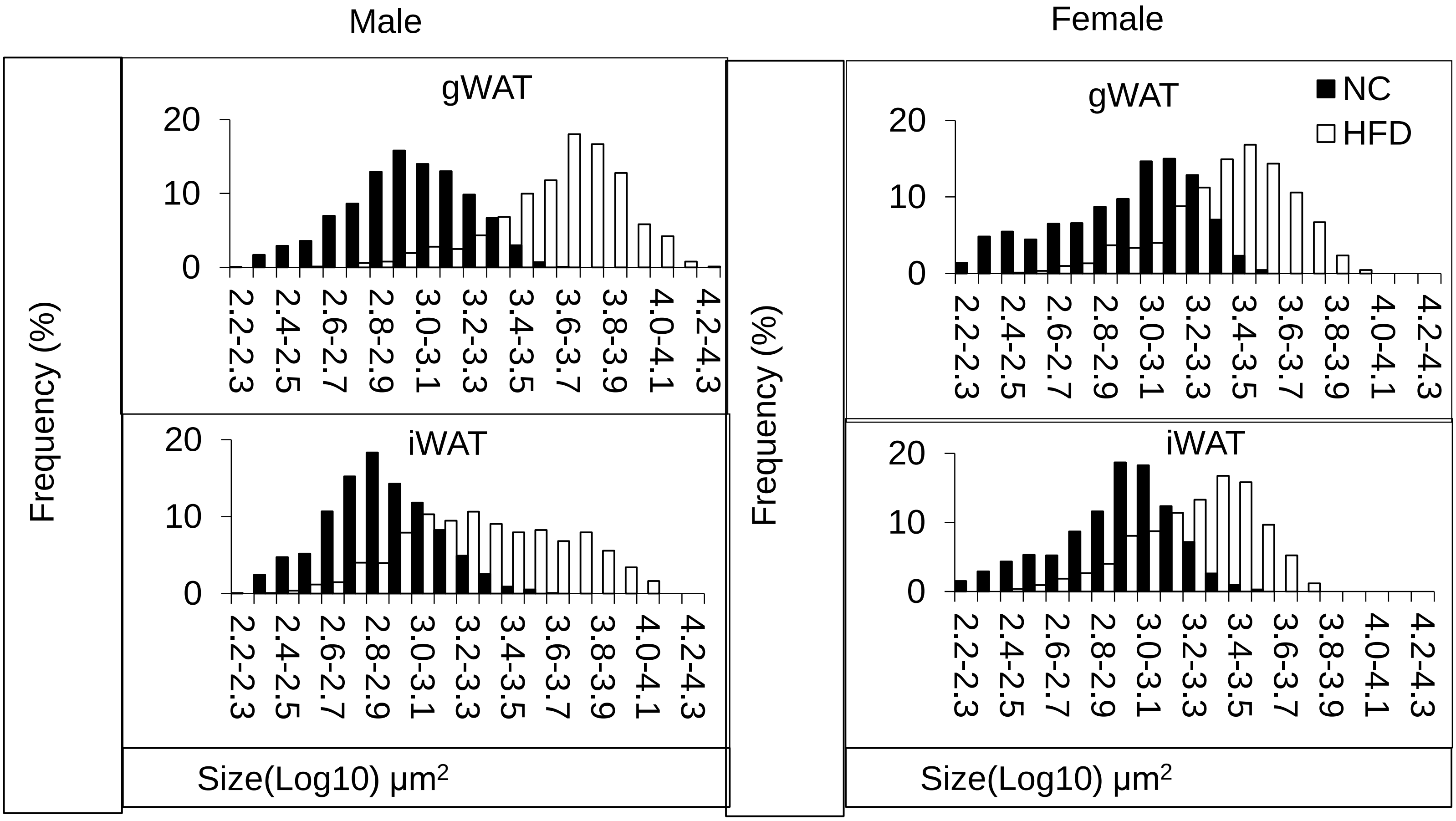

Supplementary Figure 2. Food and calorie intake in mice upon HFD feeding.

(A) Food and calorie intake in 8 weeks of HFD-fed KI/+ male and female mice compared with their littermate control NC-fed mice. Mice were started on HFD at 9 weeks of age and euthanized at 17 weeks; n = 3–5 per group. NC-fed mice consumed a lesser amount of the food than HFD-fed mice. HFD-fed male and female mice consumed an average of 15 and 14.5 g/week, respectively. NC-fed male and female mice consumed 22.3 and 18.8 g/week, respectively. However, there were no significant differences in average calorie intake either between male and female mice or between the two dietary groups (NC-fed male and female mice 75.7 and 63.8 Cal/week, respectively; HFD-fed male and female mice 8.3 and 76.7 Cal/week , respectively. **(B)** Frequency distribution of adipocyte size (area μm^2) in male and female KI/+ mice fed on either NC or HFD for 8 weeks. Mice were started on HFD at 9 weeks of age and euthanized at 17 weeks (n = 3 per group).

Supplementary Figure 3

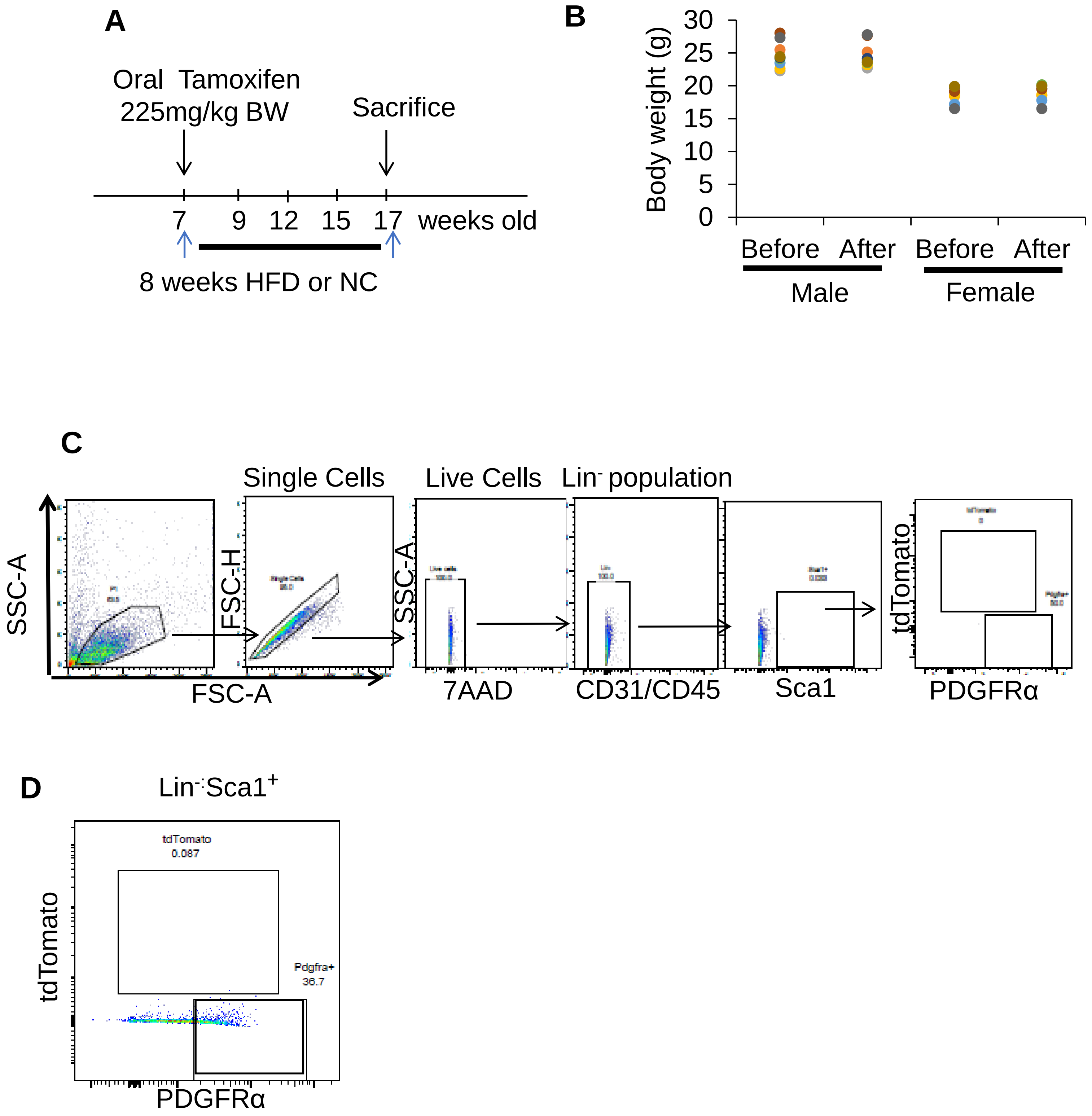

Supplementary Figure 3. Schematic diagram of TAM administration and gating strategy for flow cytometry analysis.

(A) Experimental model of KI/td mice. Tamoxifen (TAM) was administered at the age of 7 weeks. After one week of rest, these mice were fed either NC or HFD from 9 weeks to 17 weeks. Mice were euthanized at 17 weeks of age. We used the same experimental strategy for all experiments. (**B)** Body weight (g) of the KI/td mice before and after administration of TAM at 225 mg/kg BW for five consecutive days at the age of 7 weeks. Male & female, n = 12 per group. (**C)** Gating strategy for the evaluation of tdTomato^{+} APs in the Lin^{-}(CD31^{-}/CD45^{-}) Sca1^{+}PDGFRα^{+} population. (**D)** Fluorescence minus one (FMO) was used to justify the gating strategy.

Supplementary Figure 4

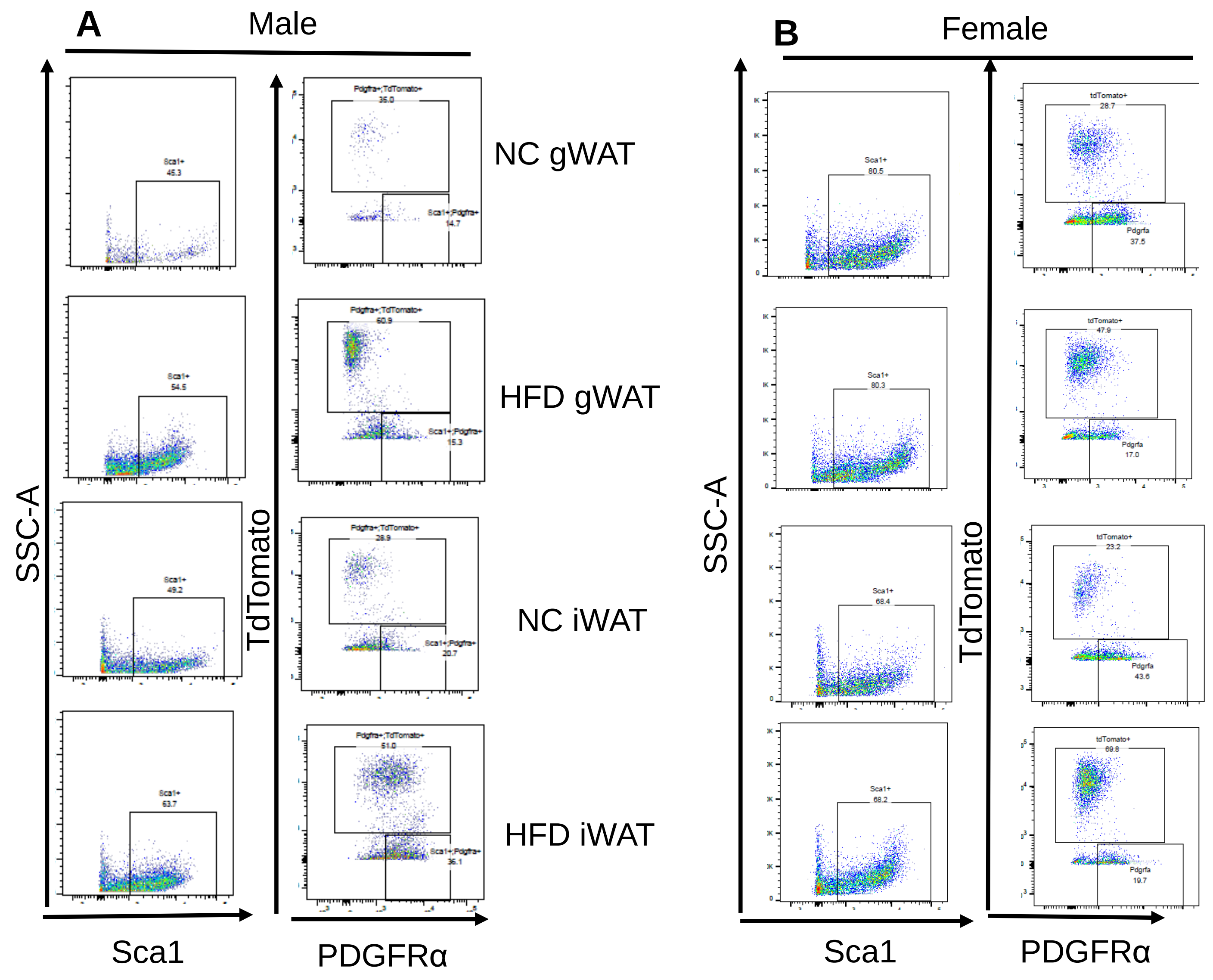

Supplementary Figure 4. tdTomato^{+} APs proliferate in response to HFD stimuli.

(**A, B**) Representative flow cytometry images of WAT depots in male and female KI/td mice fed either NC or HFD for 8 weeks. Mice were started on HFD at 9 weeks of age and euthanized at 17 weeks. (Male: NC, n = 8: HFD, n = 7; female: NC, n = 5; HFD, n = 6).

Supplementary Figure 5

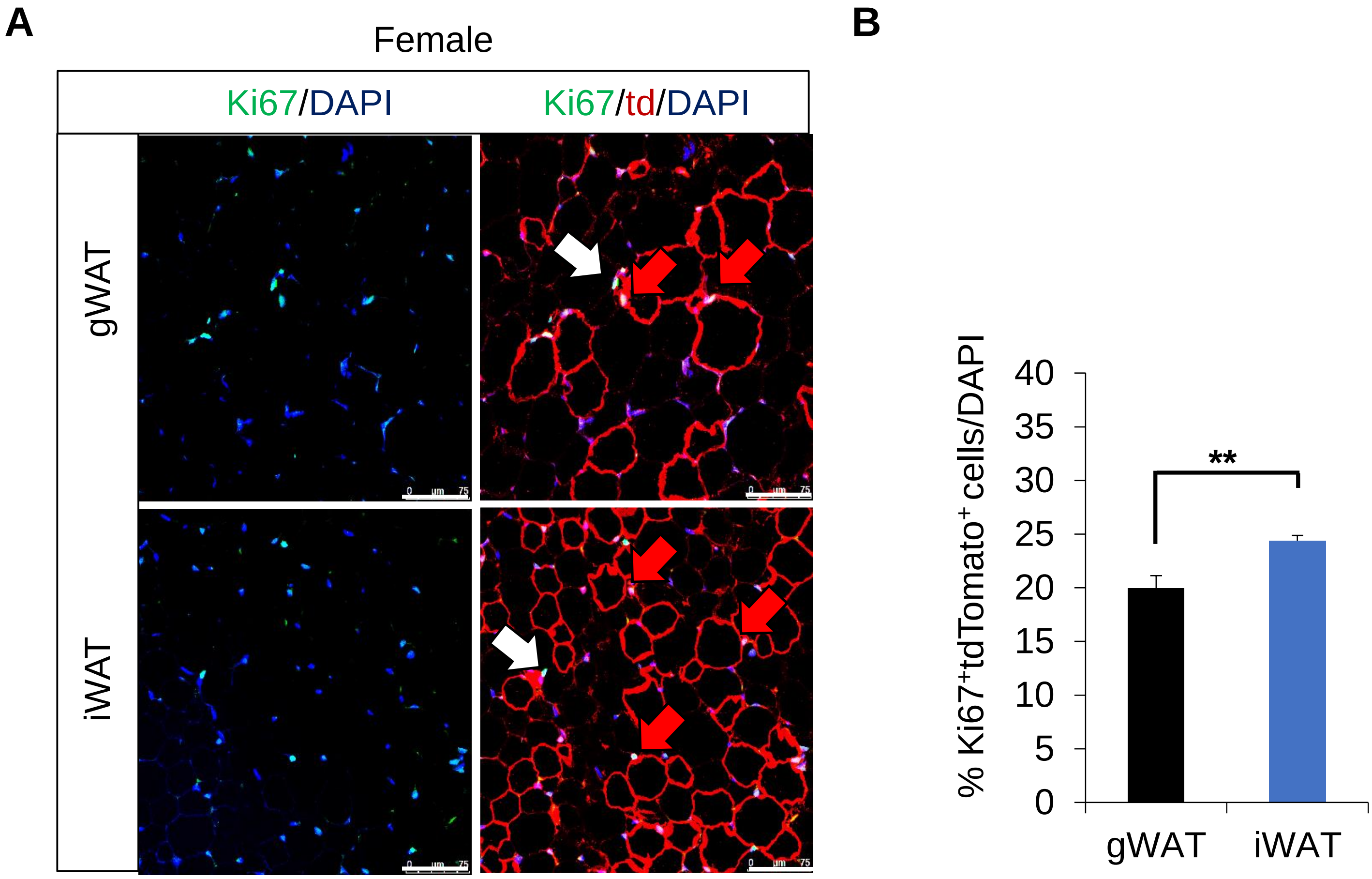

Supplementary Figure 5. Proliferative potential of newly-formed labeled APs in the iWAT of HFD-fed mice.

(A) Representative confocal images of gWAT and iWAT stained with anti-Ki67 and anti-tdTomato antibodies in KI/td female mice fed HFD for 8 weeks (n = 4). Mice were started on HFD at 9 weeks of age and euthanized at 17 weeks. Scale = 75 μm. White and red arrows indicate $Ki67^{+}$ and $Ki67^{+}/tdTomato^{+}$ cells. **(B)** Quantification of $Ki67^{+}$ cells and $Ki67^{+}/tdTomato^{+}$ cells/DAPI in both depots of HFD-fed KI/td female mice. Data represent mean ± SEM. Statistical analysis was performed using Student's *t*-test (*$p < 0.05$, **$p < 0.01$).

Supplementary Figure 6

A

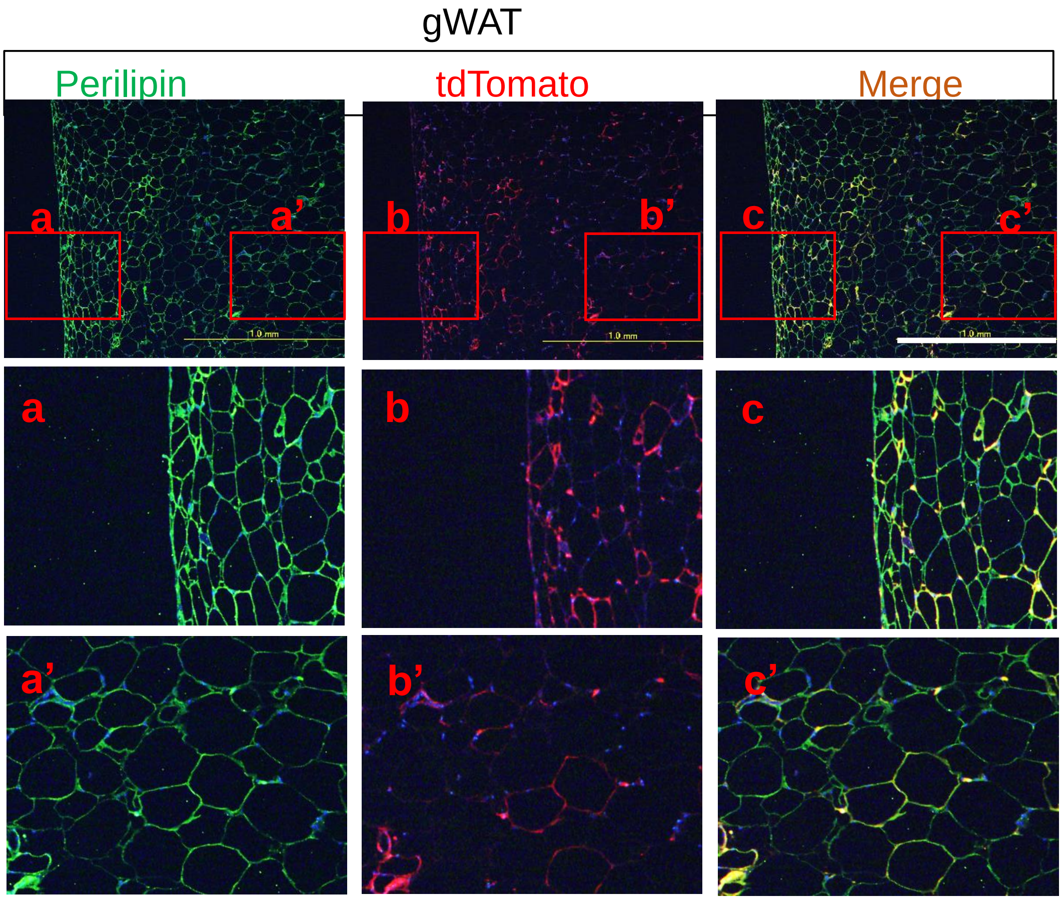

B

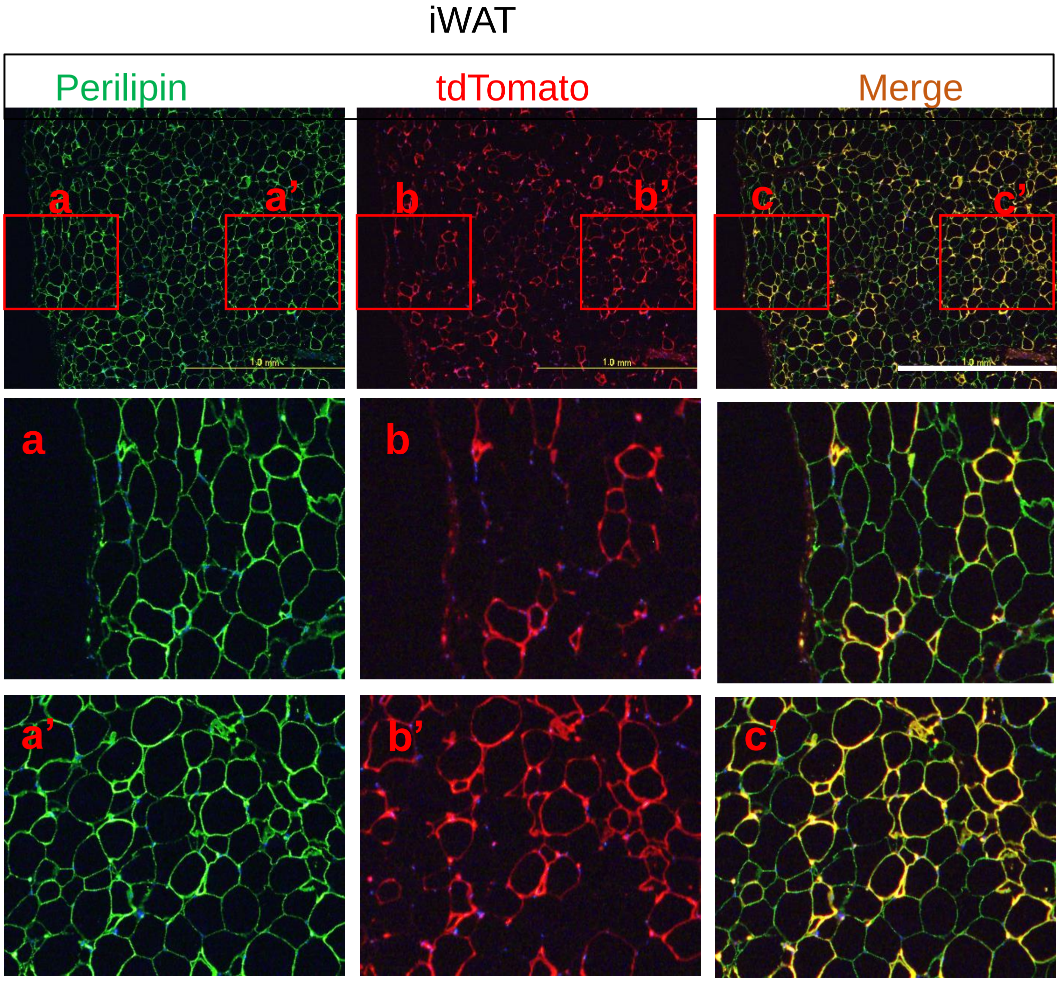

Supplementary Figure 6. HFD induces the generation of smaller adipocytes in the iWAT of male mice.

(A, B) Representative images of the gWAT and iWAT of male KI/td mice fed HFD for 8 weeks, stained with anti-perilipin (green) and anti-tdTomato (red) antibodies. Scale = 1 mm (n = 5). These data reveal that iWAT contains more smaller adipocytes than gWAT (gWAT contains larger adipocytes in the deeper layers of the tissue than in the outer layer). Mice were started on HFD at 9 weeks of age and euthanized at 17 weeks.

Supplementary Figure 7.

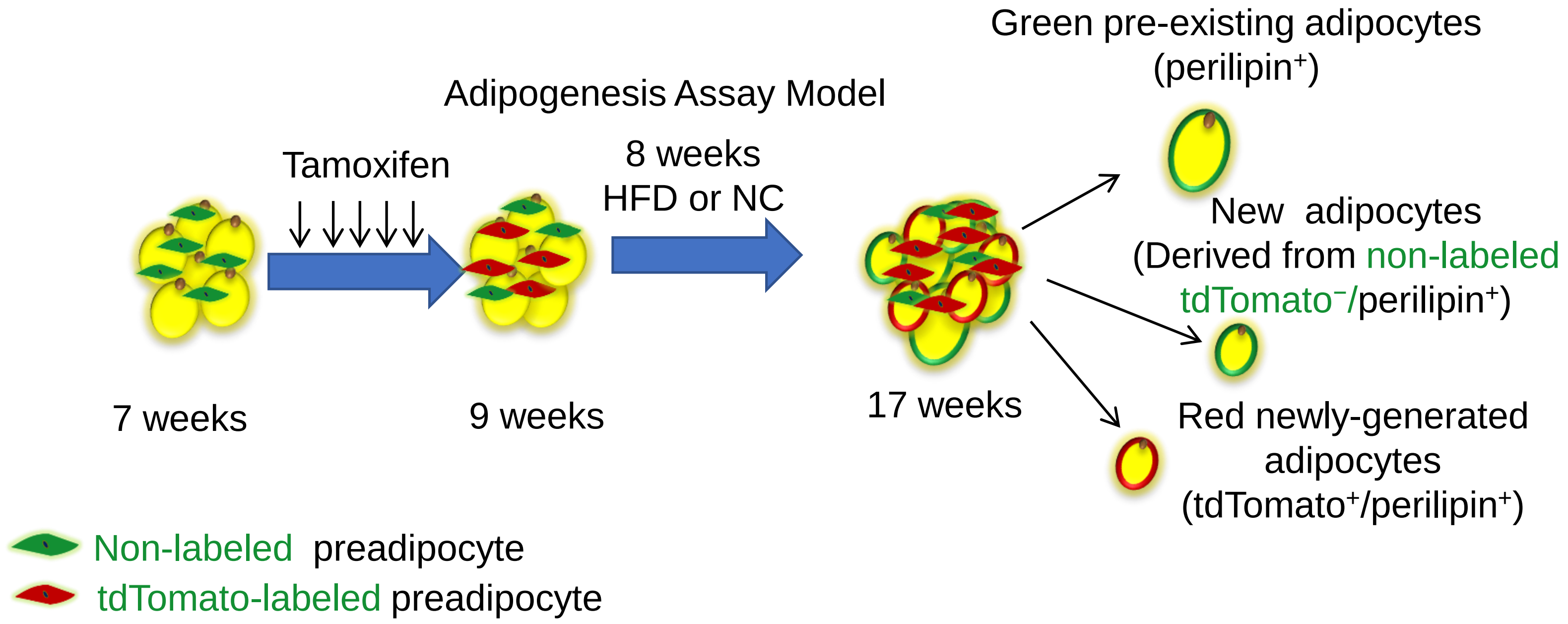

Supplementary Figure 7. *De novo* adipogenesis assay model.

Proliferation and differentiation of PDGFRα^{+} APs upon HFD-feeding in the KI/td mice model. Tamoxifen (TAM) was administered at the age of 7 weeks. Mice were started on HFD at 9 weeks of age and euthanized at 17 weeks.

Supplementary Figure 8.

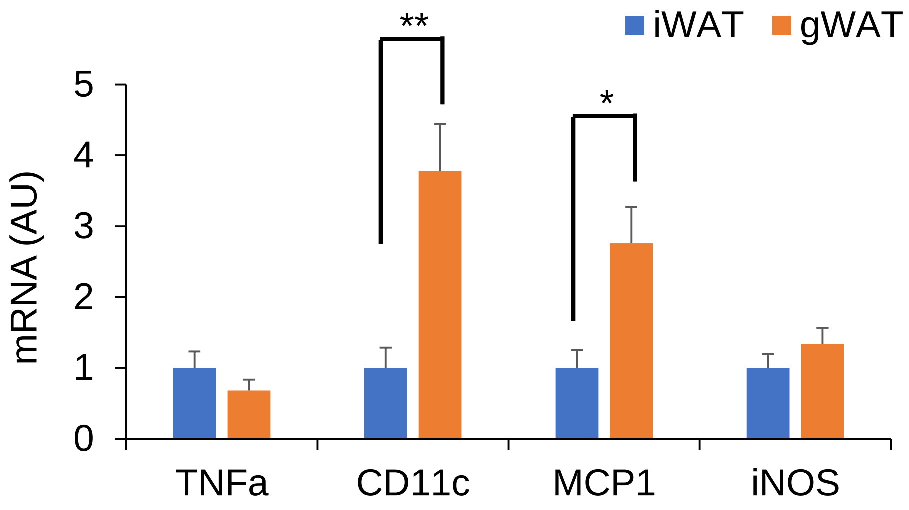

Supplementary Figure 8. iWAT is less inflammatory than gWAT.

mRNA expression levels of inflammatory marker genes in WAT depots of KI/td male mice fed HFD for 8 weeks (n = 5). Mice were started on HFD at 9 weeks of age and euthanized at 17 weeks. Data represent mean ± SEM. Statistical analysis was performed using Student's *t*-test; (*$p < 0.05$, **$p < 0.01$).
