# Supplementary material for: Fate of adipocyte progenitors during adipogenesis in mice fed a high-fat diet
Source: Mol Metab. 2021 Sep 23;54:101328. doi: 10.1016/j.molmet.2021.101328 (PMC8495176; doi:10.1016/j.molmet.2021.101328)
Supplement: Supplementary file 2 — Multimedia component 2 [file mmc2.pdf]

TABLE 1

**REAGENT or RESOURCE**	**SOURCE**	**IDENTIFIER**
Antibodies		
Anti-GFP guinea-pig polyclonal antibody	Frontier Institute Co. Ltd.	Cat#GFP-GP-Af1180
Anti-rabbit perilipin	Santa Cruz Biotechnology	Cat#Sc-67164
Anti-goat td-tomato polyclonal antibody	SICGEN Antibodies	Cat#AB8181-200
Anti-RFP rabbit monoclonal antibody	Rockland	Cat#200-301-379
Anti-rabbit Ki67 antibody	Abcam	Cat#ab15580
APC anti-mouse CD140α	BioLegend	Cat#135907 (Clone: APA5)
APC anti-mouse IgG2a, κ isotype control	eBioscience	Cat#17-4724-81
APC/Cy7 anti-mouse Ly-6A/E (Sca-1)	BioLegend	Cat#108125 (Clone: D7)
APC/Cy7 anti-mouse IgG2b κ isotype control	BioLegend	Cat#400523
PE-Cy7 anti-mouse CD31	eBioscience	Cat#25-0311-81 (Clone: 390)
PE-Cy7 anti-mouse IgG2a, κ isotype control	eBioscience	Cat#25-4031-81
PE-Cy7 anti-mouse CD45	eBioscience	Cat#25-0451-82 (Clone: 30-F11)
Anti-mouse Purified rat CD16/CD32	BD Biosciences	Cat#553141
7-AAD (PerCP-Cy5.5)	BD Biosciences	Cat#559925
Alexa Fluor® 488 conjugate goat anti-guinea pig IgG (H+L)	Life technologies	Cat#A11073
Alexa Fluor® 488 conjugate donkey anti-rabbit IgG (H+L)	Life technologies	Cat#A21206
Alexa Fluor® 568 conjugate donkey anti-goat IgG (H+L)	Life Technologies	Cat#A11057
Alexa Fluor® 555 conjugate goat anti-rabbit IgG (H+L)	Cell signaling	Cat#4413
DAPI	Molecular Probes	Cat#D1306
**Chemicals, Peptides, and Recombinant Proteins**		
Tamoxifen	Sigma-Aldrich	Cat#T5648
Sunflower oil	Fujifilm Wako Chemicals	Cat#196-15265
Collagenase from Clostridium histolyticum	Sigma-Aldrich	Cat#C6885-1G

Bovine serum albumin	Sigma-Aldrich	Cat#A8022-100G
BD Pharm lyse lysing buffer	BD Biosciences	Cat#555899
PBS(-)	Nacalai tesque	Code 14249-24
DAKO real antibody diluent	DAKO	Ref# S2022
Normal goat serum	Vector Laboratories	Ref# S-1000
Fetal bovine serum (FBS)	Gibco	Ref# 10437-028
Ethanol (99.5%)	Fujifilm WAKO Chemicals.	Cat#057-00456
4% Paraformaldehyde phosphate buffer solution	Fujifilm WAKO Chemicals.	Cat#163-20145<br>Lot# SKH3491
Direct PCR (Tail)	Viagen Biotech Incorporation	Cat#102-T
Proteinase K, recombinant PCR grade	Roche Diagnostics	Cat#03115828001<br>Lot# 48602000
TKS GflexTM DNA polymerase	Takara	Cat# R060A
Ethidium bromide	Invitrogen	Cat#15585-011
Agarose S gel	Nippon Gene	Cat#312-01193
**Critical Commercial Assays**		
Click iT ® EdU Alex Flour® 555 Imaging Kit	Molecular Probes	Ref#C10338
Isogen	NIPPON GENE	Code#311-02501<br>Lot#26000K
RNeasy mini kit (250)	Qiagen	Cat#74106
TB Green Premix Ex Taq^{TM} II (Tli RNaseH Plus)	TAKARA	Cat#RR820A<br>Lot#AK31127A
New hematoxylin solution	Muto Pure chemicals Co, limited	Lot# 131209
New eosin solution	Muto Pure chemicals Co, limited	Lot# 131028
β-Mercaptoethanol	Gibco	Cat#21985-023
**Experimental Models: Organisms/Strains**		
PDGFRα-GFP-Cre-ER^{T2} knock-in mice	(Miwa and Era, 2015)	RIKEN BRC (Tsukuba, Japan)
B6.Cg-Gt(ROSA)26Sor$^{tm9(CAG-td-Tomato)Hze}$/J	Dr. Koichi Ikuta (Kyoto University)	N/A
**Software and Algorithms**		
FlowJo version 8	FlowJo	https://www.flowjo.com/
ystat2004	ystat2004.xls for Windows	Igaku Tosho Shuppan Co., Ltd, Tokyo, Japan
ImageJ Software 11.53a	National Institute of Health, USA	https://imagej.nih.gov/ij/notes.html
**Other**		
Normal chow (NC) diet	CLEA, Japan	CE-2
HFD (60% KCAL Fat)	Research Diets	Cat# D12492<br>Lot# 20120201

Tf2b	Forward	TGGAGATTTGTCCACCATGA
	Reverse	GAATTGCCAAACTCATCAAAACT
tdTomato	Forward	ATGGTGAGCAAGGGCGAGGAGGTCA
	Reverse	TCGAACTCGTGGCCGTTCATGGAGC
Pdgfrα	Forward	AGCAGGCAGGGCTTCAACGG
	Reverse	ACACAGTCTGGCGTGCGTCC
Dpp4	Forward	TTGTGGATAGCAAGCGAGTTG
	Reverse	CACAGCTATTCCGCACTTGAA
Pi16	Forward	TGGATCTTCACAACCAGTACCG
	Reverse	CAGCTCGTCATCCCACCTC
Icam1	Forward	GTGATGCTCAGGTATCCATCCA
	Reverse	CACAGTTCTCAAAGCACAGCG
Pparγ	Forward	TTTTCCGAAGAACCATCCGATT
	Reverse	ATGGCATTGTGAGACATCCCC
Tnfα	Forward	ACGGCATGGATCTCAAAGAC
	Reverse	AGATAGCAATCGGCTGACG
Cd11c	Forward	TGTTTGAGTGTCAGGAGCAGG
	Reverse	AGGTCACCTAGTTGGGTCTTG
Mcp1	Forward	CATCCACGTGTTGGCTCA
	Reverse	GATCATCTTGCTGGTGAATGAGT
iNos	Forward	CAGCTGGGCTGTACAAACCTT
	Reverse	CATTGGAAGTGAAGCGTTTCG
